# A giant splenic hamartoma associated with hematologic disorders: A case report

**DOI:** 10.1016/j.amsu.2018.11.003

**Published:** 2018-11-16

**Authors:** Mauricio Gonzalez Urquijo, Mario Rodarte-Shade, Raul Rangel-Rangel, Jorge A. Castillo-Meraz, Jaime R. Rodriguez-Tejeda, Gerardo Gil-Galindo

**Affiliations:** a Tecnologico de Monterrey, Escuela de Medicina y Ciencias de la Salud, Dr. Ignacio Morones Prieto O 3000, Monterrey, 64710, Mexico; b Department of Surgery, Hospital Metropolitano, “Dr. Bernando Sepúlveda”, Adolfo López Mateos No. 4600, San Nicolás de los Garza, Nuevo León, 66400, Mexico; c Department of Pathology, Hospital Metropolitano, “Dr. Bernando Sepúlveda”, Adolfo López Mateos No. 4600, San Nicolás de los Garza, Nuevo León, 66400, Mexico

**Keywords:** Splenoma, Splenic hamartoma, Spleen tumor, Splenectomy

## Abstract

**Introduction:**

Splenic hamartoma is a primary benign tumor of the spleen, with approximately 150 cases documented in the literature to date, with only a few cases associated with symptoms and hematologic disorders.

**Presentation of case:**

A 49-year-old female with no past medical history, presented to the emergency department complaining of a three-month history of intermittent abdominal pain and 12 kg of weight loss. Physical examination revealed abdominal distension and a big palpable and painless mass on the left side of her abdomen measuring 14 cm. Laboratory tests were significant for anemia and thrombocytopenia, with levels of 9.7 g/dL and 47 × 10^9^/L respectively. Ultrasonography showed splenomegaly with a hypoechoic splenic mass and the computed tomography showed a 14 cm splenic mass with heterogeneous enhancement during the arterial phase. A laparotomy with splenectomy was unremarkably accomplished. Histological examination revealed abnormal red pulp proliferation and showed unorganized sinusoid-like vascular channels, compatible with splenic hamartoma. The patient was discharged on postoperative day 3 without complications. She was seen at the ambulatory clinic 6-months after the surgical procedure with a normal blood count.

**Discussion:**

Although splenic hamartoma is very rare, it must be included in the differential diagnosis of splenic mass-forming lesions. This type of tumor has some specific radiological features. However, the diagnosis of this disease must be based on clinical features and confirmed by pathology.

**Conclusion:**

In patients with splenic tumors, splenectomy is indicated in cases where malignancy cannot be excluded, when symptoms occur, or in the rare cases of consequent hematologic disorders.

## Highlights

• Case report of a 49-year-old female patient presented with abdominal pain and hematologic disorders due to a spleen hamartoma.
• Little over 150 cases have been reported in medical literature.
• Splenic hamartoma must be included in the differential diagnosis of splenic mass-forming lesions.
• Splenectomy is the treatment of choice for these lesions.

## 1 Introduction

In 1861 Rokitansky discovered a benign lesion of the spleen, known as hamartoma. Only about 120–150 cases have been described in the English literature [[1], [2], [3], [4]]. They are usually incidental findings in otherwise asymptomatic patients, with an incidence of 0.024%–0.13% [5].

These lesions are vascular proliferative neoplasms characterized by CD8 immunopositivity of the vascular endothelial lining cells [1], composed of irregularly arranged tortuous vascular channels lined by splenic sinus endothelium and separated by pulp cord-like elements [5]. Nevertheless, it is essential to differentiate this type of lesions, from malignant tumors, characterized by neovascularity, tortuosity, and displacement of adjacent splenic vessels [3].

Other terms for splenic hamartoma (SH) are splenoma, nodular hyperplasia of the spleen, tumor-like congenital malformations, splenadenoma, intrasplenic splenunculus and accessory spleen in spleen [3,5].

A minority of patients with large hamartomas have symptoms. Herein, we present a case of a relatively large palpable symptomatic splenoma in a 49-year-old female patient that presented with abdominal pain, anemia, and thrombocytopenia and that was resolved completely after a splenectomy. The work has been reported in line with the SCARE criteria [6].

## 2 Presentation of case

A 49-year-old female patient was admitted to the hospital with a 3-month history of intermittent colicky mild abdominal pain, with a mass-like distention of the left side of her abdomen, and a weight loss of 12 kg. The patient had no relevant medical history, reporting only allergy to penicillin. On examination, the patient was hemodynamically stable and afebrile. Physical examination revealed abdominal distension and a big palpable and painless mass on the left side of her abdomen measuring 14 × 14 cm, and a tympanic colonic margin to percussion. After a thorough abdominal exploration, no positive signs for peritonism were detected.

Laboratories showed a decreased hemoglobin of 9.7 g/dl, a normal white blood count of 5.4 × 10^9,^ and thrombocytopenia of 47 × 10^9^/L. No other alterations were encountered. An abdominal ultrasound was done, showing a giant, hypoechoic mass that was impossible to define.

**Fig. 1:**
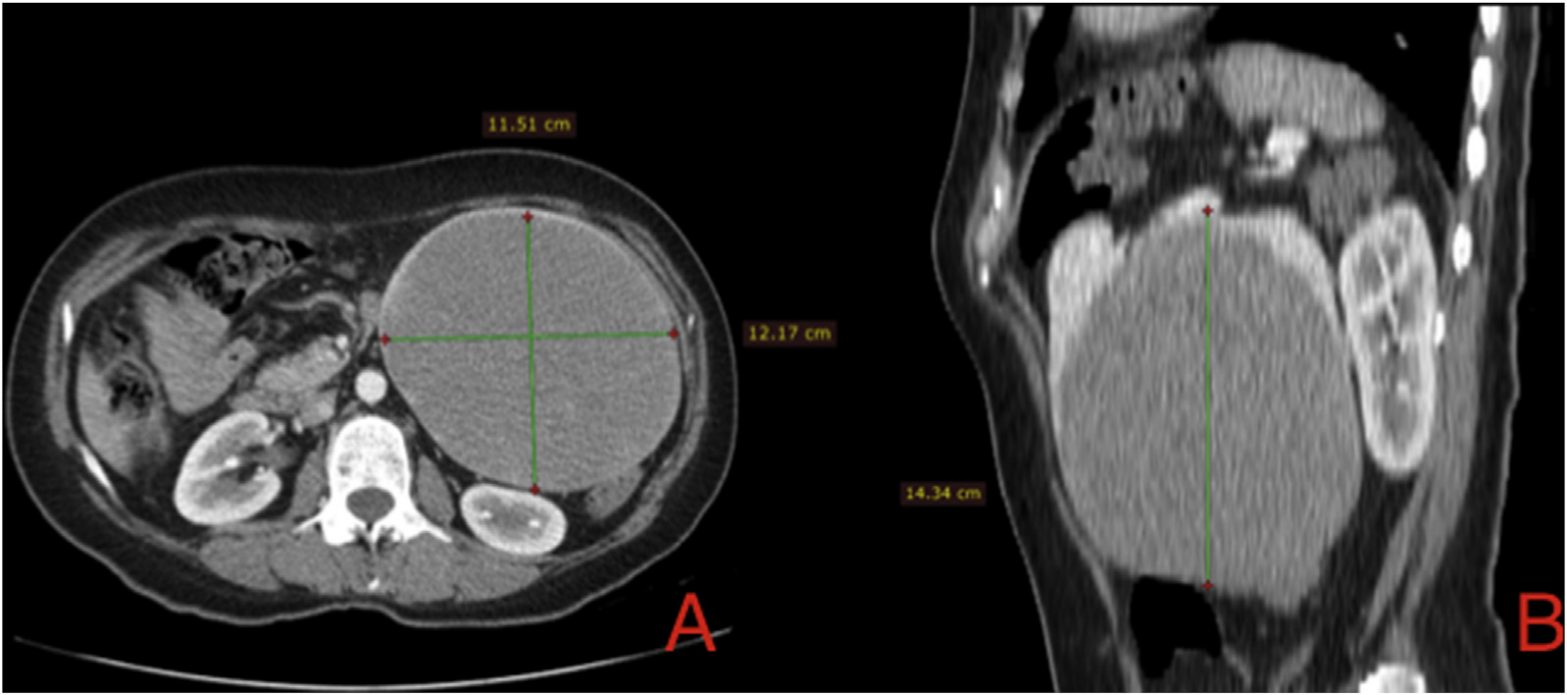
Abdominal CT **A)** Axial view with a 11.5 × 12.1 cm spleen tumor with heterogeneous enhanced on the arterial phase. **B)** Sagittal view, with the greatest dimension of the tumor of 14.3 cm.

**Fig. 2:**
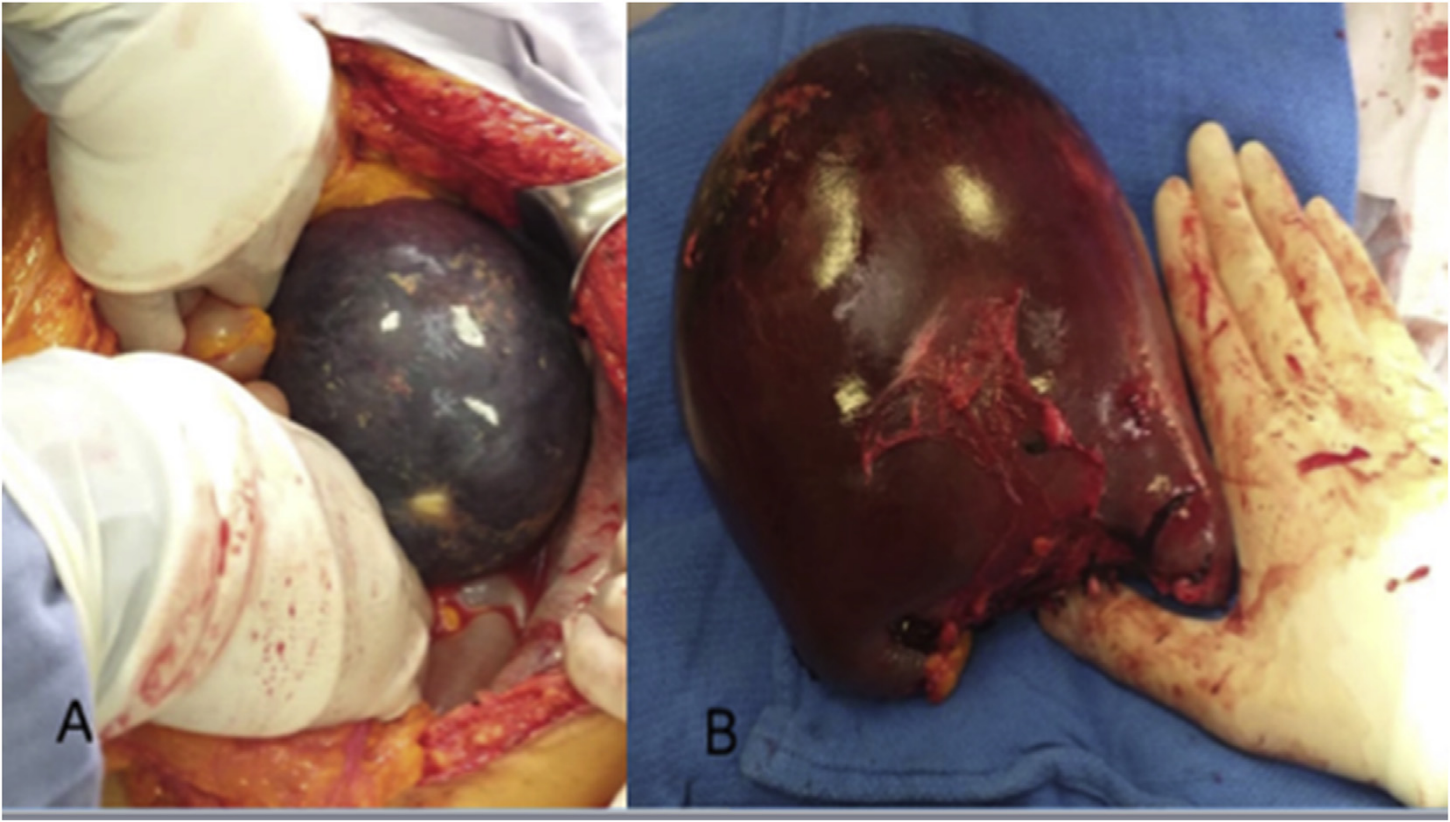
**A)** Abdominal cavity exposing the giant spleen. **B)** Spleen resected with a well-circumscribed tumor.

Abdominal computed tomography (CT) was performed, and images before the administration of contrast material showed a slightly hypodense, well-circumscribed, encapsulated mass on her left abdominal side measuring 11 × 11 × 14cm (Fig. 1, Fig. 2b). This mass was compressing the splenic flexure of the descending colon, with no obvious invasion. After the contrast bolus injection, in the arterial phase, a heterogeneous enhancement was noted on the mass. No vessel invasion was noticed. In the late venous phase, the mass became isodense.

The patient underwent an exploratory laparotomy, and after the abdominal cavity was exposed, a splenectomy was performed (Fig. 2a and b). The patient evolved favorably and was discharged on her third postoperative day.

**Fig. 3:**
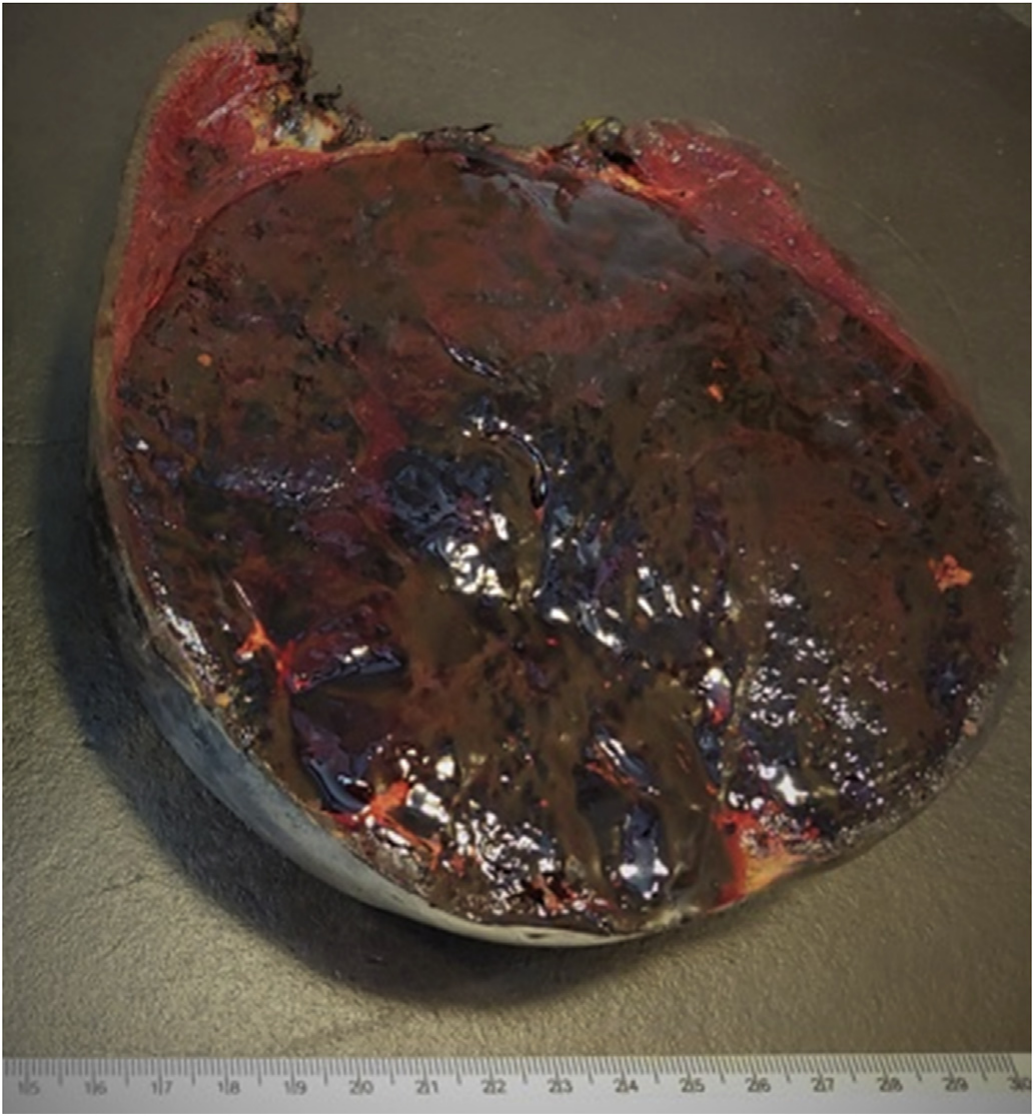
The cut surface of the resected spleen, illustrating a solitary, well-delimited protruding mass with no evidence of infiltration.

**Fig. 4:**
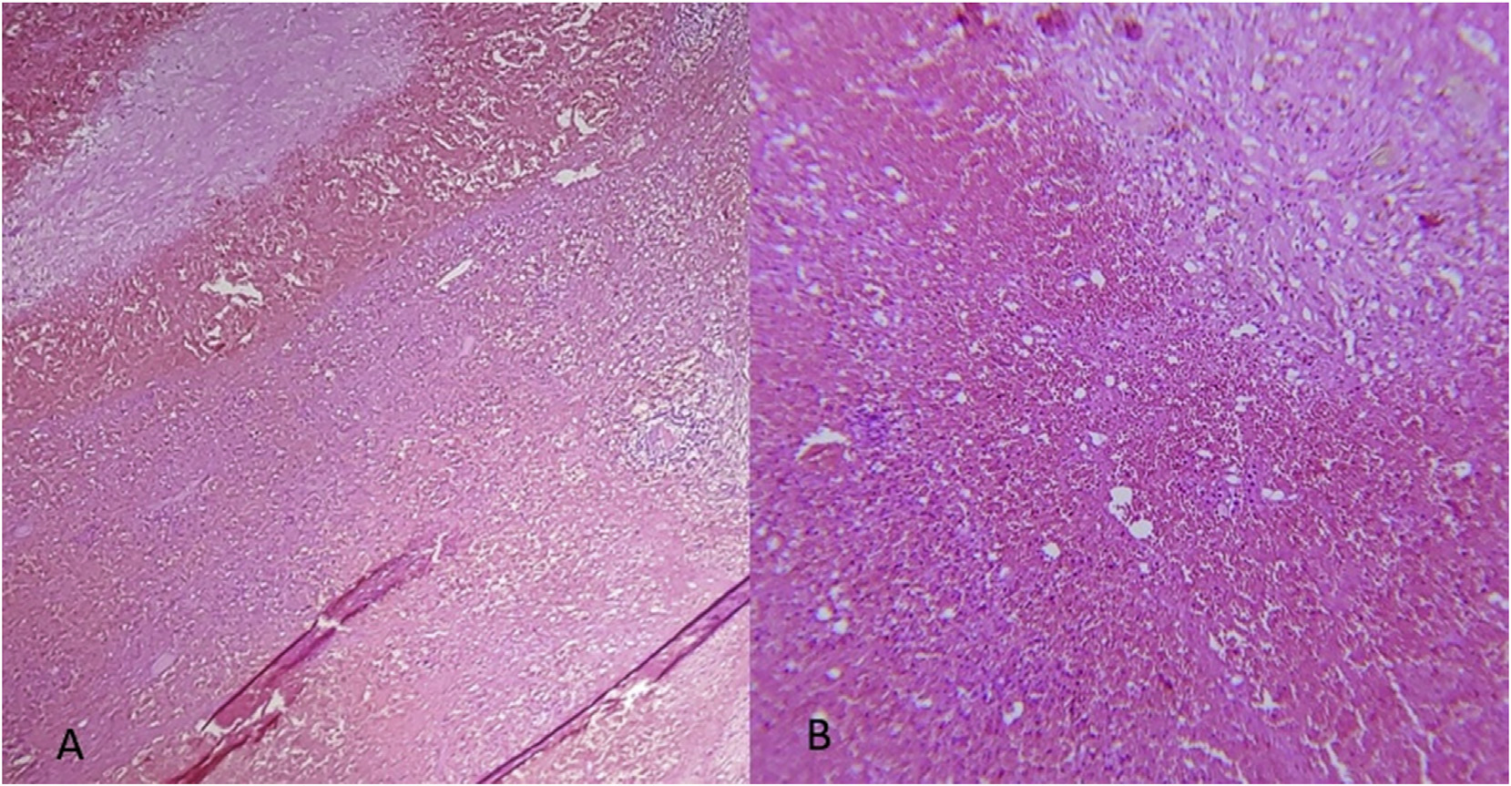
**A)** Low-power view showing unorganized sinusoid-like vascular channels. (H&E x 4). **B)** The fibrotic tracts showed no evidence of atypical cells. (H&E, x 10).

On histopathology examination, the cut surface of the resected spleen showed a solitary, well-circumscribed, bulging mass of 14 cm with no evidence of infiltration (Fig. 3). Low-power view showed unorganized sinusoid-like vascular channels, and the fibrotic tracts showed no evidence of atypical cells (Fig. 4a and b).

The combined morphologic and histologic profile supported a diagnosis of splenic hamartoma. The patient was seen at the ambulatory clinic six months after her surgery with an auspicious evolution, presenting normal hemoglobin and platelet levels.

## 3 Discussion

Patients with SH have been reported with a wide age range between 5 months and 86 years, with a mean of 47 years [2,7] and with no sex predilection [2,4]. This can be compared to our case, where our patient was a 49-years old female, and even though some articles in the literature state that this type of tumor tends to be larger in women because of female sex hormones [4,8] no statements can be proven.

Splenic hamartomas are rare benign lesions originating from the red or white pulp of the spleen, categorizing it in a pulposal type, and a lymphoid type, respectively. There is a third type, which is the most common type, containing elements of both types previously stated [[8], [9], [10]]. More than 80% of the cases are asymptomatic, and SHs are normally incidental findings during imaging studies, surgery, or postmortem [7].

This tumor may be symptomatic, and symptoms may be triggered due to splenomegaly, causing a feeling of weight in the left upper quadrant of the abdomen [11], thus corresponding to our patient symptoms. Splenomas may also cause anemia, thrombocytopenia, or pancytopenia, being the latter more rarely encountered [8]. Moreover, our patient presented with low hemoglobin and platelet levels, due to the important hypersplenism she presented, returning to her normal levels after her surgery was performed. Other less common symptoms include fever, night sweats, malaise, and spontaneous rupture [2,12]. Seyama et al. [13] studied the limited cases of rupture of this tumor reported in the English literature, with a tumor maximum size of 5.4 cm, a low to mild level of splenomegaly, with a maximum diameter and weight of the spleen of 15 cm and 520 g. These cases indicate that the weight and size of the spleen, or the dimension of the hamartoma, are not closely related to the rupture of this tumor.

Clinical or imaging diagnosis of this type of tumor is difficult to make before the operation, nevertheless, two documented cases succeed in having a preoperative diagnosis [5,12]. Sonographically most splenic hamartomas are hyperechoic solid masses, with or without cystic changes, being hypervascular in doppler ultrasound, making this study a more sensitive modality than CT in demonstrating the lesion [4,9,14]. In the latter, hamartomas appear as isodense or hypodense solid masses with calcifications or fatty components and may demonstrate heterogeneous contrast enhancement relative to the adjacent normal parenchyma [4,14]. This last feature appeared in our patient CT, with lack of the formers. Magnetic resonance imaging (MRI) may show isointensity on T1 -weighted images; heterogeneous hyperintensity or hypointensity on T2 weighted images [4,14]. We didn't order an MRI because of the lack of this study in our public hospital. Additionally, this study may distinguish fibrous from non-fibrous hamartomas [15]. Although SH has some clinical and image features that may suggest the type of lesion, the definitive diagnosis depends on histopathological findings [14,15].

SHs may present as solitary or multiple lesions forming round, well circumscribed, bulging nodules compacting the contiguous normal splenic parenchyma [1,10]. Our patient presented with only one giant lesion of 14 cm on his greater dimension, consequently compressing the spleen. This tumor may vary in size with a median size of 5 cm and a maximum reported size of 23cm [15].

The main differential diagnosis of this disease includes other vascular tumors, for instance splenic hemangioma, which is the most common benign tumor of the spleen, arising from sinusoidal epithelial cells, only shows reactivity for endothelial cell markers including CD31 and CD34, in contradistinction with SH, whom cells apart of showing reactivity for these markers, factor VIII-related antigen, and vimentin, also show reactivity for T-lymphocytes markers such as CD8 [4,8,12]. Littoral cell angioma is characterized by expression of both endothelial and histiocytic markers, it is associated with splenomegaly, and appears as a low attenuating lesion in contrast-enhanced CT. Lymphangioma manifests as a subcapsular nodule or as diffuse lymphangiomatosis in young patients. Hemangioendothelioma, has an intermediate histology between that of a hemangioma and an angiosarcoma, with lining cells showing an intermediate degree of atypia and the latter characterized for being a primary malignant tumor of non-lymphoid origin, plus for being highly aggressive with a poor prognosis [15]. At last, a sclerosing angiomatoid nodular transformation of the spleen, also identified as multinodular hemangioma, is altered red pulp entrapped by nonneoplastic stromal proliferation [4]. Other differential diagnoses include solid lesions of the spleen such as an inflammatory myofibroblastic tumor, disseminated fungal or mycobacterial infections, sarcoidosis, lymphoma, and metastasis of any primary tumor. These last two lesions have a dense spreading enhancement or prolonged enhancement on post-contrast CT and MRI [3,8,14]. Finally, SH can clinically mirror a lymphoproliferative disorder; however, the histology does not show Reed-Stemberg cells nor malignant lymphoid cells [16].

## 4 Conclusion

Although splenic hamartoma is very rare, it must be included in the differential diagnosis of splenic mass-forming lesions. The use of multimodal radiologic imaging may be helpful to identify the diagnosis preoperatively, with the definitive diagnosis depending on histopathologic examination, with the main purpose of differentiating this benign lesion from a malignant tumor. Splenectomy is indicated in cases where malignancy cannot be excluded, in symptomatic patients, or in the rare cases of consequent hematologic disorders.

## Consent

Written informed consent was obtained from the patient for publication of this case report and accompanying images. A copy of the written consent is available for review by the Editor-in-Chief of this journal on request.

## Ethical approval

NA.

## Sources of funding

This research did not receive any specific grant from funding agencies in the public, commercial, or not-for-profit sectors.

## Author contribution

Gerardo Gil- Galindo: He helped with the final version of the manuscript, and with the final edition.

Jorge Castillo: He helped with the study concept, and with the images of radiology and pathology, along with their interpretations. He also helped with the presentation of the case.

Jaime Rodriguez Tejeda: He is the attending pathologist who helped diagnosed the case. He helped with the histology images, and he helped with the writing of the manuscript.

Mauricio Gonzalez Urquijo: He is a third year general surgery resident. He was the leader of the work, he design the case report. He recollected data, and wrote the manuscript.

Mario Rodarte Shade: He is the general surgery program director, he helped with the manuscript and the final version of it.

Raul Rangel: He is a general surgery attending who helped with the case, and with the data collection.

## Conflicts of interest

None.

## Please state any conflicts of interest

None.

## Research registration number

NA.

## Guarantor

Mauricio Gonzalez Urquijo.

## Provenance and peer review

Not commissioned, externally peer reviewed.

## Appendix A Supplementary data

SCARE Guideline Checklist _spl_2_spl SCARE Guideline Checklist _spl_2_spl
